# The risk of secondary sex ratio imbalance and increased monozygotic twinning after blastocyst transfer: data from the Japan Environment and Children’s Study

**DOI:** 10.1186/s12958-019-0471-1

**Published:** 2019-02-22

**Authors:** Hiromitsu Hattori, Akane Kitamura, Fumiaki Takahashi, Norio Kobayashi, Akiko Sato, Naoko Miyauchi, Hidekazu Nishigori, Satoshi Mizuno, Kasumi Sakurai, Mami Ishikuro, Taku Obara, Nozomi Tatsuta, Ichiko Nishijima, Ikuma Fujiwara, Shinichi Kuriyama, Hirohito Metoki, Nobuo Yaegashi, Kunihiko Nakai, Takahiro Arima, Japan Environment and Children’s Study Group, Toshihiro Kawamoto, Hirohisa Saito, Reiko Kishi, Nobuo Yaegashi, Koichi Hashimoto, Chisato Mori, Fumiki Hirahara, Zentaro Yamagata, Hidekuni Inadera, Michihiro Kamijima, Ikuo Konishi, Hiroyasu Iso, Masayuki Shima, Toshihide Ogawa, Narufumi Suganuma, Koichi Kusuhara, Takahiko Katoh

**Affiliations:** 1 Department of Informative Genetics, Environment and Genome Research Center, Tohoku University Graduate School of Medicine, 2-1, Seiryo-machi, Aoba-ku, Sendai, Miyagi 980-8573 Japan; 2 Clinical Reseach, Innovation and Education Center, Tohoku University Graduate School of Medicine, 2-1, Seiryo-machi, Aoba-ku, Sendai, Miyagi 980-8573 Japan; 3 Department of Obstetrics and Gynecology, Tohoku University Graduate School of Medicine, 2-1, Seiryo-machi, Aoba-ku, Sendai, Miyagi 980-8573 Japan; 4 Tohoku Medical Megabank Organization, Tohoku University Graduate School of Medicine, 2-1, Seiryo-machi, Aoba-ku, Sendai, Miyagi 980-8573 Japan; 5 Department of Pharmacy, Tohoku University Graduate School of Medicine, 2-1, Seiryo-machi, Aoba-ku, Sendai, Miyagi 980-8573 Japan; 6 Division of Disaster Public Health, International Research Institute for Disaster Science, Tohoku University Graduate School of Medicine, 2-1, Seiryo-machi, Aoba-ku, Sendai, Miyagi 980-8573 Japan; 7 Department of Informative Genetics, Environment and Genome Research Center, Tohoku University Graduate School of Medicine, 2-1 Seiryo-cho, Aoba-ku, Sendai, 980-8575 Japan

**Keywords:** Blastocyst transfer, Secondary sex ratio, Monozygotic twins, Japan Environment and Children’s Study

## Abstract

**Background:**

Some studies have suggested that blastocyst transfer is associated with i) imbalance in the secondary sex ratio (SSR) (which favors male offspring), ii) increased incidence of monozygotic twins (MZT). In contrast, others have not found these changes. In this study, we evaluated the association between blastocyst transfer and SSR and MZT, considering potential parental confounders.

**Methods:**

The Japan Environment and Children’s Study is a large, nationwide longitudinal birth cohort study funded by the Ministry of the Environment of Japan. We used this large dataset, including 103,099 pregnancies, to further investigate the association between blastocyst transfer, SSR and MZT, using spontaneously conceived pregnancies, non-assisted reproductive technology (non-ART) treatment (intrauterine insemination and ovulation induction with timed intercourse) and cleavage stage embryo transfer for comparison. We evaluated the association with each group, the SSR, and the frequency of MZT, calculating the adjusted odds ratio (AOR) using multivariable logistic regression analyses, adjusting for potential parental confounders such as basic health and socioeconomic status.

**Results:**

For each group (spontaneous conception vs. non-ART treatment vs. cleavage stage embryo transfer vs. blastocyst transfer), the percentages of males were 51.3% vs 50.7% vs 48.9% vs 53.4% and the monozygotic twinning rates per pregnancy were 0.27% vs 0.11% vs 0.27% vs 0.99% respectively. Multivariate logistic regression analyses indicated that blastocyst transfer was significantly associated with a higher SSR and higher incidence of MZT than the other three groups (SSR: AOR 1.095, 95% CI1.001–1.198; MZT: AOR 4.229, 95% CI 2.614–6.684).

**Conclusions:**

There are significant relationships between blastocyst transfer and SSR imbalance and a higher occurrence of MZT.

**Electronic supplementary material:**

The online version of this article (10.1186/s12958-019-0471-1) contains supplementary material, which is available to authorized users.

## Background

In Japan, there has been a steady decrease in the overall neonatal secondary sex ratio (SSR) since 1970 (male/female: 1.070), with a significant decline to 1.056 in 2016 (Ministry of Health, Labour and Welfare Vital Statistics of Japan 2016) [1]. A number of biological and environmental factors related to the parents have been shown to reduce the SSR. These include older paternal age [2], factors causing metabolic alterations in women, such as diabetes [3], stressors (i.e., war, earthquakes and economic distress), toxins (i.e., smoking, pollutants and pesticides), [4–8] and regionality (race) [9].

Recent advances in sequential culture media have led to the reliable growth of embryos to the blastocyst stage. Blastocyst transfer (BT) is a well-established method employed throughout the world. BT after in vitro fertilization (IVF) and intracytoplasmic sperm injection (ICSI) has facilitated selection of top-quality embryos which results in high implantation and pregnancy rates compared with early cleavage stage embryo transfer (CT) [10]. Blastocyst culture allows selection of those embryos that have embryonic gene activation [11]. Another potential advantage of BT is better embryo-uterine environment for implantation [12]. Furthermore, since 2008, the single embryo transfer (SET) policy introduced by the Japan Society of Obstetrics and Gynecology has significantly decreased the multiple pregnancy rate [13]. In addition, BT facilitates preimplantation genetic testing (PGT) [14]. However, there remain concerns about BT and its adverse effects with regard to higher SSR and increased incidence of monozygotic twinning (MZT). Many studies have suggested that BT is associated with a male-biased SSR imbalance, compared with CT [15–20]. In contrast, other studies have shown no significant differences in the male-to-female ratio after BT [21, 22]. Some studies have also indicated higher MZT rates after BT [16, 23–25]. MZT carries additional risks, including higher perinatal mortality and morbidity, and increased risks of developmental anomalies, prematurity and discordant growth [25]. Vitthala et al. reviewed the wide variation between studies on the reported incidence of MZT after BT (1.4–13.2%) [25]. However, because of their small sample sizes, these studies did not provide sufficient information on various potential confounders such as maternal age, body mass index (BMI), smoking, and basic characteristics. There are still many unknown factors with regard to the SSR imbalance and MZT incidence.

The Japan Environment and Children’s Study (JECS) is a large, nationwide longitudinal birth cohort study directly funded by the Ministry of the Environment of Japan. In this study, we used data from JECS to investigate the association between BT and SSR and MZT, using spontaneously conceived pregnancies, non-assisted reproductive technology (non-ART) treatment (intrauterine insemination and ovulation induction/timed intercourse) and CT for comparison using multivariable logistic regression analysis.

## Materials and methods

### Study participants

Details of the JECS recruitment and sampling strategy, as well as baseline characteristics of participants, have been described previously [26]. JECS aims to evaluate the effects of environmental factors on the health and development of children from early pregnancy up to their thirteenth birthday. Subjects were recruited in 15 regions of Japan by regional centers from January 2011 to March 2014. The present study is based on the jecs-ag-ai-20,160,424 data set, which was released in June 2016. We analyzed a total of 103,099 pregnant women and 99,094 infants. All pregnant women and fathers-to-be (*n* = 50,629) provided written informed consent to participate in JECS.In the present investigation, 4673 pregnancies were excluded because of missing data on the pregnancy course, including miscarriage and stillbirth (*n* = 3922) and the method of pregnancy (*n* = 725), and missing data for classification of sex (*n* = 11) and triplet pregnancies (*n* = 15) with insufficient data (Additional file 1). When data was missing for one twin (*n* = 110), the data of both twins was excluded. Information on mode of conception and CT vs BT was obtained from chart review. We classified the pregnancies into four groups, spontaneous conception (*n* = 92,022), non-ART treatment (*n* = 3546), CT (*n* = 728), and BT (*n* = 2020). The spontaneous conception group was used as a background control and in order to study the impact of parental health and basic characteristics (age, BMI, metabolic equivalent, calorie intake, education, income, smoking status, alcohol intake status, physical and mental health condition). The non-ART treatment group was also used to study the impact of parental health and basic characteristics related to subfertility without IVF. For infants resulting from multiple pregnancies (*n* = 1556), we confirmed the MZT using doctors’ records of ultrasound scan diagnosis in early pregnancy. A viable pregnancy was defined as the presence of a heartbeat confirmed by transvaginal ultrasound in early gestation, and the number of fetuses and gestational sacs was assessed. MZT was diagnosed when more than one fetus with a heartbeat was seen in the same gestational sac, either in the same amniotic sac (monochorionic/monoamniotic) or in two amniotic sacs separated by a septum (monochorionic/diamniotic). The placentation information was included in the data set, and the diagnoses of placentation were based on ultrasound and delivery data.

The study does not include preimplantation genetic screening or diagnosis cycles.

This study was approved by the Ethics Committee of the National Institute for Environmental Studies, the Core Center of JECS and the Ethics Committee of Tohoku University School of Medicine on 10th December 2015 (reference number 2015–1-559). Written informed consent was obtained from all participants.

### Basic characteristics

Data on parental health and basic characteristics were collected using medical records and self-administered questionnaires. We obtained information on age, BMI (pre-pregnancy), physical and mental health summary scores, psychological distress, and metabolic equivalent (physical activity level per day). In particular, alcohol consumption and smoking habits were recorded following the diagnosis of pregnancy. The collected data included details of socioeconomic factors such as education and family income as well as the subjective health and mental conditions assessed by the medical outcomes study 8-Item Short-Form Health Survey (SF-8 score) [27] and the 6-item Kessler psychological distress scale (K6) [28]. The SF-8 score is a health survey that provides a health-related quality of life (QOL) profile consisting of 8 short items, and the K6 is a screening survey method for psychological distress for the general population which involves 6 questions about the subject’s emotional state. Both methods are the most widely used worldwide in health surveys. Low family income, defined by the annual revenue of the household, was categorized as an annual income of 4,000,000 Japanese yen or less. Low-education level for a couple was defined as not having advanced beyond high school academically. Maternal past histories of hyperlipidemia, diabetes, hyperthyroidism and hypothyroidism were examined using doctors’ records.

### Data analysis

All statistical analyses were performed using SAS version 9.4 (SAS Inc., Cary, NC). Basic characteristics of the four groups were compared using the chi-squared test for categorical values and one-way analysis of variance (ANOVA) for numerical continuous variables. We calculated the adjusted odds ratio (AOR) using multivariable logistic regression analyses to evaluate the heterogeneity of basic characteristics between the four groups and the association between the method of conception and SSR and frequency of MZT. The adjusted variables included basic maternal characteristics, i.e., age, BMI, metabolic equivalent, calorie intake, education, income, smoking status, alcohol intake status, physical and mental health condition (SF-8 score; physical health condition and mental component summary score), psychological distress (K6 score) and maternal past medical history (e.g., hyperlipidemia, diabetes, hyperthyroidism, and hypothyroidism). We further adjusted for selected basic paternal characteristics (age, BMI, education, smoking, alcohol intake, SF-8 score and K6 score) as described above. These factors were selected since they could be regarded as potentially confounding factors. We then evaluated the association between the method of pregnancy and SSR and MZT rates. Missing values were imputed by the fully conditional specification method. Two hundred data sets were created with missing values replaced by imputed values based on a model that incorporated all variables. The final result then combined these 200 individual results by applying Rubin’s rule [29]. A two-sided *P* <  0.05 was regarded as statistically significant.

## Results

### Demographic and basic characteristics

Background data on the subjects in the spontaneous conception, non-ART treatment, CT and BT groups are presented in Table 1 and Additional file 2. The mean parental ages were higher in the CT and BT groups than in the spontaneous conception and non-ART treatment groups. The mean BMI was significantly higher in the parents of the non-ART treatment, CT, and BT groups compared with the spontaneous conception group. The levels of both maternal and paternal education were highest in the non-ART treatment group (*P* <  0.0001). The metabolic equivalent, calorie intake, physical health condition and K6 scores were lower for mothers in the CT and BT groups than for those in the other groups, unlike the mental component summary score, which was higher. The proportion of parents with a smoking habit when pregnancy was diagnosed was lower in the CT and BT groups compared with the spontaneous conception group. Consumption of any amount of alcohol at least once a month as recorded at the time pregnancy was diagnosed was lowest in the mothers of the CT and BT groups. However, there was no significant difference in alcohol consumption habits, physical health condition, mental component summary and K6 in fathers. Maternal past histories of diabetes, hyperthyroidism and hypothyroidism were highest in the CT and BT groups and hyperlipidemia were more prominent in the non-ART treatment, CT and BT groups than in the spontaneous conception group. There was no significant difference between the CT and BT groups for any basic characteristic except family income and mental component summary.

**Table 1:** Characteristics of the study subjects according to the method of pregnancy

Characteristics	Spontaneous conception	Non-ART treatment	CT	BT	Spontaneous vs Non-ART treatment vs CT and BT
Mother (Pregnancies) (n)	92,022	3546	728	2020	
Age (year)	30.4 ± 5.0	32.7 ± 4.2	35.6 ± 4.0	35.6 ± 3.9	<.0001
BMI (kg/m2)	21.2 ± 3.3	21.4 ± 3.6	21.3 ± 3.0	21.3 ± 3.1	<.0001
Metabolic equivalent/min	414.1 ± 732.9	334.8 ± 604.6	256.0 ± 488.1	258.6 ± 462.5	<.0001
Calorie intake	385.4 ± 729.3	318.0 ± 597.4	235.0 ± 453.0	249.5 ± 551.8	<.0001
Low education (%)	37.3	22.4	21.9	22.8	<.0001
Smoking habit (%)	18.9	8.5	4.4	4.8	<.0001
Alcohol consumption habit (%)	65.6	63.2	60.6	62.4	<.0001
Physical component summary (SF-8 score)	45.3 ± 5.3	44.1 ± 7.6	43.7 ± 7.9	43.8 ± 8.2	<.0001
Mental component summary (SF-8 score)	46.1 ± 7.3	46.9 ± 7.1	47.6 ± 7.2	47.0 ± 7.0	<.0001
Assessing depression (K6 score)	3.7 ± 3.9	3.2 ± 3.4	2.8 ± 3.0	3.0 ± 3.4	<.0001
Hyperlipidemia (%)	0.5	1.3	0.5	0.7	<.0001
Diabetes (%)	0.2	0.4	0.5	0.5	<.0001
Hyperthyroidism (%)	1.0	1.3	1.4	1.8	0.001
Hypothyroidism (%)	0.9	2.1	3.4	3.9	<.0001
Family					
Low income (%)	41.5	26.8	16.7	20.2	<.0001
Father (n)	47,208	1929	410	1082	
Age (year)	32.4 ± 5.8	34.4 ± 5.2	37.2 ± 5.4	37.6 ± 5.5	<.0001
BMI (kg/m2)	23.4 ± 3.5	23.5 ± 3.4	23.9 ± 3.2	23.8 ± 3.3	<.0001
Low education (%)	45.1	31.3	30.3	32.3	<.0001
Smoking habit (%)	51.2	38.7	33.8	33.6	<.0001
Alcohol consumption habit (%)	78.8	78.0	78.1	80.1	NS
Physical component summary (SF-8 score)	51.0 ± 5.3	51.1 ± 5.3	51.2 ± 4.7	50.9 ± 5.3	NS
Mental component summary (SF-8 score)	49.8 ± 6.1	49.9 ± 6.2	50.0 ± 6.2	50.1 ± 5.9	NS
Assessing depression (K6 score)	2.6 ± 3.5	2.5 ± 3.4	2.3 ± 3.2	2.5 ± 3.5	NS
Infants (n)	92,545	3696	751	2102	
Male (%) (95% CI)	51.3 (49.2–53.4)	50.7(48.6–52.8)	48.9 (45.3–52.5)	53.4 (51.3–55.5)	NS
Sex ratio	1.05	1.03	0.96	1.15	
Pregnancies (n)	92,022	3546	728	2020	
Single (%)	99.4	95.8	96.8	95.9	<.0001
Multiple (%)	0.6	4.2	3.2	4.1	
[Monozygotic twinning (%)] (95% CI)	0.27 (0.23–0.30)	0.11 (0–0.22)	0.27 (0–0.65)	0.99 (0.56–1.42)	<.0001

Data are shown as mean ± SD (The percentages are drawn from available data and the total number varies for each category because of missing values and refer to the Additional file 2.). Smoking and alcohol consumption habits at pregnancy recognition, SF-8 score of 50 or less. Low education: defined by the highest academic background and categorized as graduation from high school or less, Low income: defined by the annual revenue of the household and categorized as an annual income of 4,000,000 Japanese yen or less. The *P* values are listed simply to give an indication of the magnitude of the inter-group differences. It is not intended that all those *P* values should have the customary probabilistic interpretation

*BMI* (body mass index), *CT* (early cleavage-stage embryo transfer), *BT* (blastocyst transfer), *NS* (not statistically significant)

### Associations between BT and the SSR

The results of multivariate logistic regression analysis showed that BT had a significant association with higher SSR (OR, 1.095; 95% CI, 1.001 to 1.198; *P* = 0.047) (Table 2). Regarding the adjusted variables, higher SF-8 scores for physical component summary and mental component summary were significantly related to a higher SSR. (*P* < 0.001, *P* = 0.030) (Additional file 3). On the other hand, a lower SSR was observed if the fathers had a history of smoking (*P* = 0.029) (Additional file 3). However, SSR in the non-ART treatment and CT groups was slightly lower or showed no difference compared with the spontaneous conception group.

**Table 2:** Adjusted odds ratios of male infants by method of fertilization and stage of embryo transfer in ART cycles

	AOR	95% CI	*P*-value
Spontaneous conception	1.000 (reference)		
Non-ART treatment	0.979	(0.915–1.048)	NS
CT	0.914	(0.789–1.058)	NS
BT	1.095	(1.001–1.198)	0.047

Adjusted for the questionnaire results for mother’s age, BMI, metabolic equivalent/min, calorie intake, education, income, smoking status, alcohol intake status, SF-8 physical component summary, SF-8 mental component summary, K6 and history of hyperlipidemia, diabetes, hyperthyroidism, hypothyroidism and the questionnaire results for father’s age, BMI, low education, smoking status, alcohol intake status, SF-8 physical component summary, SF-8 mental component summary, K6

*CT* (early cleavage-stage embryo transfer), *BT* (blastocyst transfer), *AOR* (adjusted odds ratio), *CI* (confidence interval), *NS* (not statistically significant)

### Incidence of MZT after BT

Of 778 multiple pregnancies, 274 were monochorionic. The numbers of pregnancies in the four groups were 248 spontaneous, 4 non-ART treatment, 2 CT, and 20 BT. We compared the MZT rates in the four groups (Table 1 and Additional file 2). The MZT rate in the BT group was the highest (0.99%; 20/2020), at approximately 3.7 times higher than that in the spontaneous conception group (0.27%; 248/92022). In contrast, the MZT rate after CT was similar to that of the spontaneous conception group.

As with the SSR, BT significantly increased the risk of MZT compared with the other three fertilization methods as shown by the results of multivariate logistic regression analyses (OR, 4.229; 95% CI, 2.614 to 6.844; *P* < 0.001) (Table 3). Higher K6 and lower physical health condition scores in mothers led to a lower risk of MZT (Additional file 4). Although we also examined the relationship between the increased incidence of MZT and SSR imbalance, there was no significant difference in SSR imbalance between the MZT group (51.8% [284/548]) and the non-MZT group (51.3% [50,513/98,546]).

**Table 3:** Adjusted odds ratios of MZT by method of fertilization and stage of embryo transfer in ART cycles

	AOR	95% CI	*P*-value
Spontaneous conception	1.000 (reference)		
Non-ART treatment	0.426	(0.158–1.149)	NS
CT	1.167	(0.287–4.740)	NS
BT	4.229	(2.614–6.844)	< 0.001

Adjusted for the questionnaire results for mother’s age, BMI, metabolic equivalent/min, calorie intake, education, income, smoking status, alcohol intake status, SF-8 physical component summary, SF-8 mental component summary, K6 and history of hyperlipidemia, diabetes, hyperthyroidism, hypothyroidism and the questionnaire results for father’s age, BMI, low education, smoking status, alcohol intake status, SF-8 physical component summary, SF-8 mental component summary, K6

*CT* (early cleavage-stage embryo transfer), *BT* (blastocyst transfer), *AOR* (adjusted odds ratio), *CI* (confidence interval), *NS* (not statistically significant)

## Discussion

With the development of the currently available culture media, blastocyst transfer has increasingly become a common ART treatment. BT allows for the evaluation and selection of high-quality embryos for transfer. In addition, SET helps prevent multi-zygotic multiple pregnancies. However, adverse effects of BT such as an SSR imbalance and an increase in MZT rate have been reported [15–20]. The basic characteristics of ART patients differ from those of the general population. Whether the variations in the SSR and MZT rate are a result of environmental and biological background of ART patients or BT per se is debatable.

In this study, parental basic characteristics, mean BMI and levels of education, both maternal and paternal, were higher in the non-ART treatment, CT, and BT groups compared with the spontaneous conception group. This may be related to the advanced age of patients receiving ART. In addition, the low proportion of parents with a smoking habit, lower calorie intake, lower levels of alcohol consumption, and higher mental component summary of mothers in the CT and BT groups may indicate higher levels of health consciousness of ART patients. As a demographic difference that changes sex ratio, older parental age has been shown to reduce the SSR [2]. One possible biological explanation for the decrease in sex ratio with parental age is age-related hormonal changes such as an increase in female gonadotrophin and decline in male testosterone concentrations with age [30]. It is considered that such age-related hormonal changes may affect differential mortality of XX and XY fetuses and skewed ratio of Y- and X chromosome spermatozoa [31]. Regarding the MZT, the recent review indicated that although some studies showed an increase in the MZT rate in cases of young maternal age, it can be speculated that the true underlying mechanism might be the oocyte age, or even the quality of the oocyte, rather than maternal age itself [32].

Some reports have suggested that psychological disorders such as depression are associated with the SSR [33, 34]. Regarding association between SSR and parental stress, it is indicated that the variation in the SSR may result from alteration in the sex-selective preimplantation embryo loss [35]. As mechanisms affecting this alteration, sperm abnormality, reduced coitus and perturbations in the female reproductive tract caused by stress before preconception are considered [36, 37]. Similar reasons may affect the incidence of MZT. Multivariate logistic regression analyses indicated that higher K6 and lower physical health condition scores in mothers led to a lower risk of MZT. Miscarriage caused by stress might be related to the results, but it is not known whether MZT and maternal stress are related.

Analyses also showed that maternal ongoing histories of diabetes, hyperthyroidism and hypothyroidism were highest in the CT and BT groups, and hyperlipidemia was higher in the non-ART treatment, CT, and BT groups than in the spontaneous conception group. These differences in basic characteristics are related to the fertility rate.

Multivariate logistic regression analysis of parental characteristics found a significant difference in sex ratio from blastocyst transfer compared with spontaneous conception groups (*P* = 0.047). However, based on the CI of 1.001–1.198, the difference barely achieving statistical significance, which is concerning in the setting of multiple comparisons done in the analyses for this study. Meanwhile, we found that BT caused a significant increase in MZT; results that coincided with previous reports [16, 23–25]. However, there was no significant difference in the SSR or MZT rate among the spontaneous conception, non-ART treatment, and CT groups.

There are thought to be several possible reasons for the higher SSR after BT. First is the selection of embryos for transfer based on morphological assessment by the embryologist. More male blastocysts may be selected for transfer because the cleavage of male embryos is faster than that of female embryos up to the blastocyst stage and sequential media may be preferential for male embryos [16, 38–40]. However, some studies have reported different results [21, 22]. The second issue is the timing of embryo transfer. Extended culture of embryos up to the blastocyst stage may well alter the properties of the cell surface and/or adhesiveness of embryos at implantation [41]. BT results in enhanced implantation rates, which may cause selection in favor of male embryos [19]. However, it remains unclear which factor of BT is involved in the risks of SSR.

We concluded that BT significantly increased the MZT rate, consistent with previous reports [16, 23–25]. The causes suggested in previous studies to explain the observed increase in the MZT rate are as follows: 1) extended time in culture, 2) culture medium composition, and 3) embryology laboratory experience [42–45]. The blastocyst undergoing prolonged culture might experience excess environmental stress, weakening cellular adhesion and increasing MZT, but in shorter culture, embryos might be able to tolerate such circumstances [46]. Recently, it has been reported that the risk of MZT after BT has declined significantly [46, 47]. The reason for this is unclear, but it may be because of improvements in culture media. Assisted hatching (AH) is often performed in frozen thawed ET. It is reported that AH could lead to hatching defects of the embryo, and through these defects, an inner cell mass may be pinched off and divide, forming MZT [43, 47, 48]. However, Nakasuji et al. suggested that AH and maternal age did not significantly affect the incidence of MZT [49]. Further studies are needed to confirm the findings in large series.

A strengths of this study is due to the fact that, using a population-based data set with comparison to a large number of spontaneous pregnancies and adjustment for biological and environmental factors. However, there are some limitations to our study. Firstly, it had no specific information on infertility type, the number of embryos transferred, embryo morphology, fertilization methods, or the history of ART treatments. We confirmed that the subjects had not been pregnant with twins more than twice. However, family history of twin pregnancy was not included in the questionnaire item. Secondly, the analysis of monozygotic twinning being based on very few cases after CT, should be investigated further. Thirdly, we could not differentiate between fresh embryo transfer (ET) and frozen-thawed ET, however, In Japan, frozen-thawed embryos are predominantly used (71.2%) [50]. Fourthly, and most importantly, methodological limitations must also be considered with regard to the diagnosis of zygosity using early ultrasound data. Our definition of monozygotic is limited. Fingerprinting and DNA polymorphism analyses are necessary to distinguish dichorionic diamniotic monozygotics of the same sex; however, such analyses were not performed in this study.

In conclusion, we examined the slight risks of SSR imbalance and a high incidence of MZT after BT and found little influence of parental biological and environmental factors. At present, the number of infants born as a result of ART, particularly BT, continues to increase in developed countries, including Japan. Although JECS is a large longitudinal cohort study on children’s health and development from early pregnancy up to their thirteenth birthday, we recommended further longitudinal follow-up studies are still needed to shed light on the benefits and risks of BT.

## Additional files

Additional file 1: Flowchart to identify the study population. Among the 98,426 pregnancies, those for which data on sex of the infant and the method of pregnancy were missing were excluded. Pregnancies show the number of mothers. Subjects were classified into four groups: spontaneous conception, non-ART treatment, CT and BT. CT: early cleavage-stage embryo transfer, BT: blastocyst transfer. (PDF 162 kb)

Additional file 2: The total number with data available for all parameters according to the method of pregnancy. CT (early cleavage-stage embryo transfer), BT (blastocyst transfer), smoking and alcohol consumption habits at pregnancy recognition, SF-8 score of 50 or less. Low education: defined by the highest academic background and categorized as high school graduation or lower, Low income: defined by the annual revenue of the household and categorized as an annual income of 4,000,000 Japanese yen or less. (XLS 47 kb)

Additional file 3: Odds ratios of male infants by method of fertilization and parental characteristics. CT (early cleavage-stage embryo transfer), BT (blastocyst transfer), smoking and alcohol consumption habits at pregnancy recognition, SF-8 score of 50 or less. Low education: defined by the highest academic background and categorized as high school graduation or lower, Low income: defined by the annual revenue of the household and categorized as an annual income of 4,000,000 Japanese yen or less. (XLS 42 kb)

Additional file 4: Odds ratios of MZT by method of fertilization and parents’ conditions. CT (early cleavage-stage embryo transfer), BT (blastocyst transfer), smoking and alcohol consumption habits at pregnancy recognition, SF-8 score of 50 or less. Low education: defined by the highest academic background and categorized as high school graduation or lower, Low income: defined by the annual revenue of the household and categorized as an annual income of 4,000,000 Japanese yen or less. (XLS 45 kb)

## Abbreviations

ANOVA: Analysis of variance
AOR: Adjusted odds ratio
ART: Assisted reproductive technology
BMI: Body mass index
BT: Blastocyst transfer
CT: Cleavage-stage embryo transfer
ICSI: Intracytoplasmic sperm injection
IUI: Intrauterine insemination
IVF: In vitro fertilization
JECS: The Japan Environment and Children’s Study
MZT: Monozygotic twins
OI: Ovulatory induction
PGT: Preimplantation genetic testing
SAS: Statistical analysis system
SD: Standard deviation
SET: Single embryo transfer
SSR: Secondary sex ratio
