# Supplementary figures and images for: The risk of secondary sex ratio imbalance and increased monozygotic twinning after blastocyst transfer: data from the Japan Environment and Children’s Study
Source: Reprod Biol Endocrinol. 2019 Feb 22;17:27. doi: 10.1186/s12958-019-0471-1 (PMC6387559; doi:10.1186/s12958-019-0471-1)

**Figure 1**

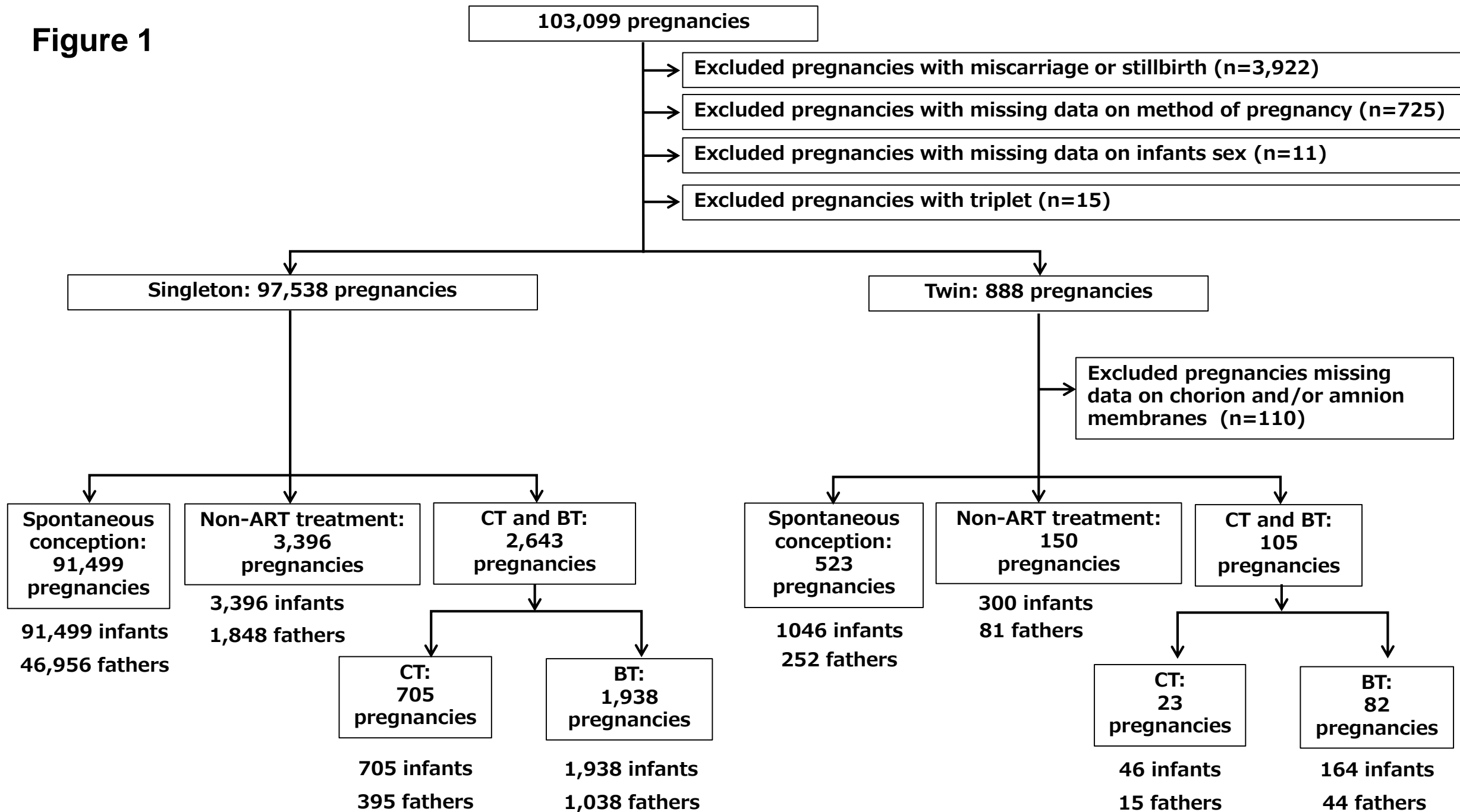

Supplement: Supplementary file 1 — Flowchart to identify the study population. Among the 98,426 pregnancies, those for which data on sex of the infant and the method of pregnancy were missing were excluded. Pregnancies show the number of mothers. Subjects were classified into four groups: spontaneous conception, non-ART treatment, CT and BT. CT: early cleavage-stage embryo transfer, BT: blastocyst transfer. (PDF 162 kb) [file 12958_2019_471_MOESM1_ESM.pdf]
